# Inclusive survey design: MCMLA's lessons learned from an inclusivity and climate assessment

**DOI:** 10.5195/jmla.2025.2159

**Published:** 2025-10-23

**Authors:** Margarita Shawcross, Brenda Linares, Emily Vardell, Jennifer Brady, Yumin Jiang

**Affiliations:** 1 maggie.shawcross@unco.edu, University of Northern Colorado; 2 blinares@umkc.edu, Associate Dean of Library Services, University of Missouri at Kansas City, Kansas City, MO; 3 evardell@emporia.edu, Associate Professor, School of Library and Information Management, Emporia State University, Emporia, KS; 4 jbrady@sbuniv.edu, Head Librarian, Zalk Veterinary Medical Library, University of Missouri, Columbia, MO; 5 yumin.jiang@cuanschutz.edu. Department Head, Collection Management, University of Colorado Anschutz Medical Campus. Aurora, CO

## Abstract

In Fall 2019, the Midcontinental Chapter of the Medical Library Association (MCMLA) welcomed a new incoming chair who outlined four priorities for their tenure including “adopting Diversity & Inclusion (D&I) values, policies, and practices in every aspect of the organization” [1]. These priorities led to the MCMLA Executive Committee approving the creation of the Diversity and Inclusion (D&I) Task Force. The task force created a survey to capture the makeup of the current MCMLA membership, as well as to assess the diversity climate of the organization.

The MCMLA D&I Task Force consulted professional resources and modified an existing survey from the Mid-Atlantic Chapter of the Medical Library Association (MAC-MLA) to develop their assessment tool. The updated survey was designed to examine several issues related to climate, such as perceptions of the diversity of MCMLA membership and leadership, participants' experiences of discrimination at MCMLA conferences, and individuals' desire to learn more about advocacy. The task force planned to use the survey results to better understand and address climate issues specific to diversity and inclusion within MCMLA. The survey was distributed to membership first in 2020 as a short survey, then in 2021as a improved, longer survey whose results were presented at our 2021 MCMLA Conference Meeting. This commentary will detail how the task force carefully designed the survey questions to incorporate timely and sensitive approaches to assessing demographics and resource needs related to diversity and inclusion.

## LEARNING ABOUT CHAPTER DIVERSITY

The need to diversify the library workforce has led numerous libraries, including public, academic, and special libraries as well as library associations to adopt diversity initiatives and policies to create more inclusive library environments. The Mid-Atlantic Chapter (MAC) was the pioneer chapter of MLA in bringing diversity to the list of their priorities [2]. Two years before MLA started their diversity initiative, MAC of the MLA was looking into ways to promote diversity within MAC membership and decided to develop and distribute first a short survey, then followed by a longer and more comprehensive survey, to get an accurate picture of the diversity of MAC as well as gather ideas to assist with programming and recruitment [2]. In 2019, the Midcontinental Chapter of the Medical Library Association (MCMLA) D&I task force spearheaded another initiative. Brenda Linares, the previous MAC Diversity Task Group Chair, started in a new position in a state within the MCMLA region and brought her experience and ideas which were then able to be used for the MCMLA Diversity Survey. The task force administered an online survey to determine the demographic makeup of the association as well as to seek members' opinions on MCMLA's efforts to address and educate on topics of diversity, equity and inclusion [3]. Where many of the original questions from the MAC Diversity Survey were included, the committee made deliberate decisions to eliminate specific questions. The committee sent out a shorter initial survey and did not have much response. The task force met later and decided re-evaluate the survey, developing a more comprehensive and inclusive survey, as well as deciding to provide incentives for participation to increase participation.

Being able to have someone who had been involved with MAC-MLA and could share her experience with the process of developing a survey, as well as the final questions sent to their membership, was helpful. The MCMLA D&I Task Force was able to save time by not having to develop a survey from scratch. As the task force reviewed the existing survey, the new MCMLA chair Shandra Knight would often ask the “so what” question, encouraging the task force to consider what would be done with the information if collected, leading to pre-existing questions being eliminated and the order of the questions being changed for flow. One of the items the task force worked to keep at the forefront was to ask questions about how members felt as part of MCMLA, as we do not have control over other environments, such as work or home-life.

Questions were reordered on the survey based on the level of importance determined by the task force. We decided to ask the climate questions first and then the demographic questions, so we had people's responses about MCMLA and did not communicate the feeling that we just cared about their demographics. The task force wanted participants to feel included and that their opinions about MCMLA and diversity were important. The original MAC survey asked if participants were treated with respect by other members of MAC, as well as if they were being treated equitably. MCMLA chose to ask questions about how respondents were treated and if they felt they were treated equally regardless of the various identities in our chapter. We collected this data using multiple-choice questions and a Likert scale (a Likert scale assumes that the strength/intensity of an attitude is linear, i.e., on a continuum from strongly agree to strongly disagree) to evaluate participants' perceptions of how MCMLA and its members treat individuals based on various identities." We concluded the survey by asking what MCMLA could do to improve diversity and inclusion within the chapter.

### Inclusive Language Around Sexual Orientation and Gender Identity

Since the questions' initial development by MAC, there have been expansions in inclusivity language as we are always learning more about the spectrum of diversity, equity, and inclusion, with most of the evolving discussions centering on the Sexual Orientation and Gender Identity (SOGI), race/ethnicity, and disability questions. The MCMLA task force discussed the purpose of including gender in a survey. The task force believed in the “so-what” purpose of this question rested in the foundation of affirmative action initiatives. For instance, men still earn more than women in the library administrative sector. ALA statistics tell us “though most library directors are women, the percentage of directors who are men exceeds the percentage of librarians who are men. Also, men's salaries tend to be higher than women's, even for the same position” [4].

Another goal for the MCMLA D&I Task Force was to better serve LGBTQIA+ members, ensuring they feel welcomed and accepted. Wanting to avoid outdated, inaccurate, or harmful terms—and recognizing that LGBTQ+, LGBTQIA, and LGBT+ are more inclusive than the now-outdated “LGBT”—the MCMLA D&I Committee carefully reviewed the research to choose terminology that reflects current, respectful, and inclusive language. The research defined sexual orientation as who you are attracted to and want to have relationships with, and who you feel romantically, sexually, and emotionally attracted to [5]. It has typically included the labels such as: gay, lesbian, straight, and bisexual, which is different from gender identity. Gender identity–defined as one's internal sense of being a man, woman, both, neither of these, or something else–is a powerful determinant of one's lived experience [6]. Gender identity can be consistent with or different from the sex that someone was assigned at birth. Sex assigned at birth is typically based on external genitalia, and is recorded as female, intersex, or male. Transgender is an umbrella term for people whose gender identity differs from the sex assigned to them at birth, while cisgender is a term for people whose gender identity aligns with their sex assigned at birth. “Nonbinary” is an umbrella term for gender identities that are not exclusively man or woman; rather, they could be a blend of both or neither. Other words that people use for nonbinary identities include agender, bigender, gender-expansive, or genderqueer. Table 1 outlines the considerations and actions taken by the D&T Task Force members to create a survey with inclusive and current terminology.

**Table 1:** Considerations and choices made to strive for inclusive language in the D&I survey

Key Consideration	Action Taken	Reasoning
Inclusive Terminology	Used LGBTQ+, LGBTQIA, LGBT+ instead of LGBT	LGBT is considered outdated
Survey Response Options	Reduced from 10 to 6	Removed pansexual, queer, and questioning/not sure
Additional Options	Added “Prefer not to say” and “Prefer to self-describe”	To respect respondent preferences
Terminology Sensitivity	Defined all terms used in the survey	To recognize ambiguity and generational differences
Heterosexual Label Update	Changed to “Heterosexual/Straight”	To modernize language
Gay and Lesbian Categories	Combined into one	To simplify and be more inclusive
Avoiding Negative Connotations	Did not use “homosexual”	To recognize its negative connotation
Use of “Queer”	Removed from options but acknowledged its complexity	Empowering to some, pejorative to others

### Inclusive Language Around Race and Ethnicity

The next intentional “so what” category the task force sought to capture demographic information about the MCMLA population was race/ethnicity. Like individuals who belong to a sexual minority group, those in a racial minority face disparities such as health, employment, and environmental. Like terms used for SOGI, terms used to refer to racial and ethnic groups continue to change and evolve over time. To have a responsive survey, it is important to use the racial/ethnic categories that the participants use themselves whenever possible, allowing for personal preference or an updated preferred designation as some designations are considered outdated. Race refers to the physical differences seen in groups or cultures that are considered socially significant, where ethnicity refers to the shared cultural characteristics such as language, beliefs, and practices. The U.S. Census Bureau considers race and ethnicity two separate things. The Census Bureau allows individuals to report themselves as: White; Black or African American; Asian; American Indian and Alaska Native; Native Hawaiian and other Pacific Island; or some other race. Respondents can report multiple races. Ethnicity on the other hand refers to a group of people that share a common descent, history, and/or homeland [7]. And because Hispanic/Latinos may be of any race(s), the U.S. Census groups Latinos or Hispanics further breaks down the ethnic group “Hispanic/Latino” into races as well. Race is considered a social construct that it is not consistently acknowledged across cultures. In an effort to be more culturally inclusive, MCMLA chose to include three categories in the survey-ethnic background, nationality, and race [8].

### Inclusive Language Around Disability Status

The task force was once again faced with the issue of why they were collecting data when they looked at asking MCMLA members if they had a disability. The original MAC survey asked participants if they had no disability, prefer not to answer, limited cognitive or physical condition or other. Worldwide, about 15% of the world population and about 12.6% of the US population have a disability [9]. The American Community Survey defines disability as having hearing difficulty, cognitive difficulty, ambulatory difficulty, self-care difficulty, or independent living difficulty [10]. Our professional ethics, as expressed by the American Library Association, also make it clear that meeting the needs of disabled patrons and workers should be made a priority [11]. The task force concluded that asking members to disclose if they had a disability (as was done in the MAC survey) was not inclusive of the disabilities that have been increasingly recognized as disabilities in recent times. For example, “depression, a mood disorder that is marked by varying degrees of sadness,” would not fall under the two main disabilities listed on the survey question as is [12]. The task force determined their “so what” for asking about disabilities was to better understand the MCMLA membership conference spaces' accessibility needs, specifically mobility for in-person conferences and audiovisual needs for both in-person and virtual. A suggestion was made for the task force to change the wording from asking an individual that self identifies with a disability how it impacts their daily lives to instead providing a blank box for folks to fill in with their needs. Therefore, the task force decided to reformat the disability question in the following way: Do you have a disability that impacts your interactions with MCMLA? If yes, tell us how you experience the impact and tell us what we can do to improve accessibility.

The open-ended responses in our survey provided feedback on the pilot survey itself that will aid us in refining our survey questions should we choose to administer the survey again in the future. In addition, the qualitative feedback collected may assist others interested in developing similar surveys. The collected data included in this article focuses on feedback on the survey itself rather than other aspects collected by the survey to provide guidance for other groups seeking to conduct similar surveys of their membership.

## LESSONS LEARNED

When collecting data and sending surveys, we realized that there would be things that worked well and things that did not. Here are some of the lessons learned for people who might be interested in conducting their own survey in the future.

One major takeaway was learning that providing fewer options specifically as it pertained to the SOGI questions was more inclusive than providing more options. We reduced the number of options that had been offered originally on the MAC survey and added an “other” option with an area for participants to enter in their preferred method to describe themselves. We've found that the terms people prefer to describe themselves often go beyond the standard response options typically used in surveys. If we don't allow respondents the space to identify themselves more accurately, we risk misrepresenting the communities we aim to understand—and may unintentionally reinforce existing social inequalities.

As for survey design though the original Diversity Survey from MAC had been created using Qualtrics, we chose to use Redcap since that was the tool in which we had access that had the level of data security we wanted. As we collected data and tried to analyze the results, we found that the task force members were not versed in Redcap, and it was difficult to learn as you go. Therefore, we recommend that one of your team members be familiar with the tool being used for gathering the data. However, the ability to privatize the data is essential, and therefore, if you must choose between familiarity of a tool over data security, we will always recommend prioritizing data security. When considering improving the usability of the survey, we chose to use conditional IF/THEN logic, so follow-up questions only appeared when relevant, minimizing screen clutter. A visible progress bar was also added to help participants track their completion status, and an easy-to-find exit button provided a sense of control and accessibility throughout the process.

When first creating a diversity survey, the D&I task force recommends taking the time to establish the purpose, or your “so what,” and establish a guideline for what the data can and will be used for, including who will have access to the data, where it will be stored, for how long, etc. As with any other survey that involves humans, make sure to disclose those guidelines to participants so they know how their information is being used, where it is being kept, and that they have the option to opt out of participation at any time. If you are offering your participants an incentive to participate, establish what the incentive will be, who will fund it, how incentives will be awarded etc. ahead of time so that all information can be disclosed from the very beginning. Make sure to send reminders and updates on response rates to all chapter members on a regular basis through a variety of communication methods – we used social media, email, and the chapter newsletter.

One of our questions focused on MCMLA's education efforts, asking survey participants about which areas of diversity, equity, and inclusion need to be better addressed in the educational programs offered by MCMLA. The available options included age, gender/gender identity, disability, ethnic background, nationality, perceived socioeconomic status, race, religion, and sexual orientation. We included an “Other” option where respondents could write in additional areas. One participant noted “weight/size is not a protected class, and yet 60%+ of the country is overweight or obese.” This participant's feedback encourages us and others to consider adding in weight/size as another aspect of diversity that could be captured in surveys and should be considered in educational offerings.

At the end of our survey, we had an option where respondents could offer “Additional Comments.” One participant noted, “I can't answer a lot of these the way they are written - I haven't experience[d] or observed anything in MCMLA relating to race because almost everyone I interact with or see here is of the same general background as myself… We should consult members with diverse backgrounds while avoiding pigeon-holing them or forcing them into doing a lot of extra work.” We appreciate this participant's feedback and note that this may be a limitation in the data that was collected in the survey. In any revised, future surveys, it may be necessary to consider the wording of the questions to recognize that there may be a limitation in the diversity of interactions our MCMLA members have in organizational activities.

## CONCLUSION

In designing and implementing the MCMLA Diversity and Inclusion survey, the task force was committed to approaching the work with care, intention, and a willingness to learn. By building upon the foundation laid by the MAC-MLA and thoughtfully adapting survey language and design, the task force created a tool that reflects current understandings of inclusive terminology and ethical data collection. The lessons learned throughout this process—especially around the importance of asking “so what” at each step—offer valuable insights for others seeking to assess and improve the inclusivity of their organizations. As MCMLA continues its diversity, equity, and inclusion journey, this survey serves as both a starting point for deeper conversations and a model for other chapters and associations working to create more welcoming and representative library communities.

## RESEARCHERS' POSITIONALITIES STATEMENT

The authors of this study represent a variety of personal backgrounds, including diverse races and ethnicities, sexual orientations, family structures, socio-economic levels, and professional roles. They have experience serving on DEI committees within professional associations and at their institutions. They do not, however, purport to represent all communities within the umbrella of DEI efforts as they can only speak to their own experiences and backgrounds (e.g., the authorship of this paper is comprised entirely of cisgender females and therefore cannot speak to the experiences of transgender individuals or cisgender men). The discussions laid out in this study should continue to seek to invite all to the table so more perspectives can be included.

## Data Availability

There is no data associated with this article.
